# Aspects of Newborn Screening in Isovaleric Acidemia

**DOI:** 10.3390/ijns4010007

**Published:** 2018-01-29

**Authors:** Andrea Schlune, Anselma Riederer, Ertan Mayatepek, Regina Ensenauer

**Affiliations:** 1 Experimental Pediatrics and Metabolism, Department of General Pediatrics, Neonatology and Pediatric Cardiology, Heinrich Heine University Düsseldorf, Moorenstrasse 5, 40225 Düsseldorf, Germany; 2 Department of Obstetrics and Gynecology, Hospital Altötting-Burghausen, Teaching Hospital of the Ludwig-Maximilians-Universität München, Vinzenz-von-Paul-Strasse 10, 84503 Altötting, Germany

**Keywords:** isovaleric acidemia, newborn screening, blood C5-carnitine, mild phenotype, short/branched chain specific acyl-CoA dehydrogenase

## Abstract

Isovaleric acidemia (IVA), an inborn error of leucine catabolism, is caused by mutations in the isovaleryl-CoA dehydrogenase (*IVD*) gene, resulting in the accumulation of derivatives of isovaleryl-CoA including isovaleryl (C5)-carnitine, the marker metabolite used for newborn screening (NBS). The inclusion of IVA in NBS programs in many countries has broadened knowledge of the variability of the condition, whereas prior to NBS, two distinct clinical phenotypes were known, an “acute neonatal” and a “chronic intermittent” form. An additional biochemically mild and potentially asymptomatic form of IVA and its association with a common missense mutation, c.932C>T (p.A282V), was discovered in subjects identified through NBS. Deficiency of short/branched chain specific acyl-CoA dehydrogenase (2-methylbutyryl-CoA dehydrogenase), a defect of isoleucine degradation whose clinical significance remains unclear, also results in elevated C5-carnitine, and may therefore be detected by NBS for IVA. Treatment strategies for the long-term management of symptomatic IVA comprise the prevention of catabolism, dietary restriction of natural protein or leucine intake, and supplementation with l-carnitine and/or l-glycine. Recommendations on how to counsel and manage individuals with the mild phenotype detected by NBS are required.

## 1. Introduction

Newborn screening (NBS) for organic acidurias such as isovaleric acidemia (IVA) has been a matter of debate [1,2,3]. Yet, IVA (OMIM #243500) has been included in many NBS programs, most recently in the United Kingdom [4] (Table 1).

**Figure 1:**
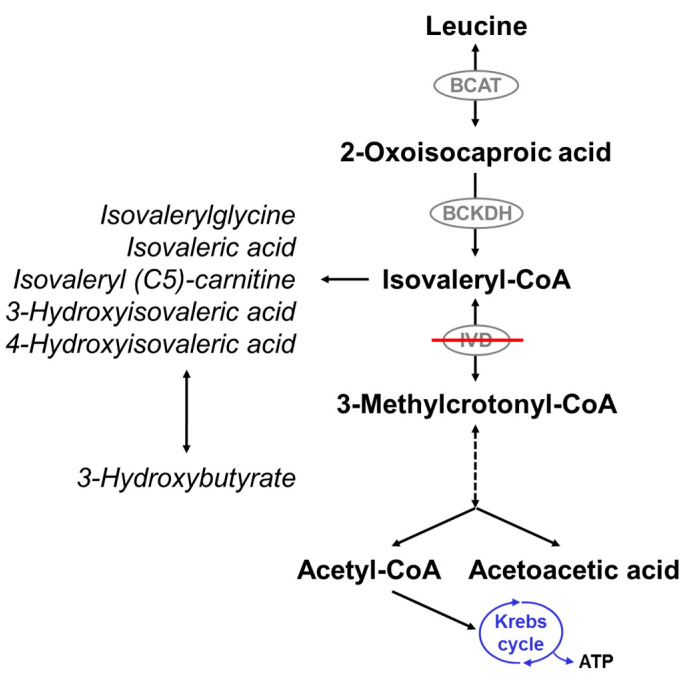
Leucine catabolism pathway. Isovaleryl-CoA dehydrogenase (IVD) catalyzes the degradation of isovaleryl-CoA to 3-methylcrotonyl-CoA. Deficiency of IVD leads to the accumulation of derivatives of isovaleryl-CoA and reduced production of acetyl-CoA and acetoacetate. BCAT: branched-chain amino acid aminotransferase; BCKDH: branched-chain alpha-ketoacid dehydrogenase.

IVA is due to a defect of isovalery-CoA dehydrogenase (IVD; Mendelian Inheritance in Man [MIM] #607036; enzyme commission [EC] 1.3.8.4), an acyl-CoA dehydrogenase (ACAD) catalyzing the third step in the intramitochondrial breakdown of leucine (Figure 1). It is caused by mutations in the isovaleryl-CoA dehydrogenase (*IVD*) gene and is inherited as an autosomal recessive trait. More than 60 disease-causing mutations in the *IVD* gene have been described. The majority are point mutations, but splice site mutations, nonsense mutations, missense mutations, deletions, and insertions have also been described [18,19,20,21,22,23,24,25,26,27,28,29,30,31,32,33].

Deficiency of IVD results in an accumulation of derivatives of isovaleryl-coenzyme A (CoA), the metabolite before the block, such as isovaleric acid, 3-hydroxyisovaleric acid, isovaleryl (C5)-carnitine, and isovalerylglycine (IVG) (Figure 1). The pathogenesis of the disease is still not fully understood. Mechanisms thought to be involved include the induction of oxidative stress through accumulating metabolites as seen in the rat brain cortex [34], the reduction of Na^+^, K^+^-ATPase activity by free isovaleric acid as shown in synaptic membranes from the cerebral cortex in young rats [35], and abnormal cellular growth signaling through activation of the mammalian target of rapamycin complex 1 (mTORC1), as suggested from studies with human IVD deficient cells [36].

The phenotypical spectrum of IVA is wide and has been further broadened by NBS. Available data suggests that early diagnosis by NBS may improve the clinical outcome of IVA, as supported by reports of less frequent relapsing episodes of metabolic decompensation [37] and short-term improvement of neurodevelopmental symptoms [37,38], even though studies on the long-term outcome of screened patients are still lacking.

Two clinical phenotypes have been observed in unscreened patients. They may become symptomatic within the first days or weeks of life, presenting with poor feeding or vomiting and severe metabolic acidosis accompanied by neurological signs including lethargy, potentially progressing to coma or death [39]. Alternatively, patients may present later in childhood with acute acidotic episodes often triggered by catabolic stress such as intercurrent illness [32,40]. In 1966, IVA was first reported by Tanaka and coworkers [41], who described two siblings of preschool age with recurrent episodes of vomiting and lethargy and an unusual odor of “sweaty feet”, in whom a massive urinary excretion of isovalerylglycine and other metabolites of isovaleryl-CoA were detected using gas chromatography (GC) and mass spectrometry (MS) [42].

Besides, a third distinct phenotype of IVA has been identified by NBS [30]. Individuals show a less pronounced accumulation of isovaleric acid and its derivatives than clinically detected patients and present with a potentially asymptomatic phenotype. So far, no severe metabolic crises have been reported in these subjects. A certain missense mutation, c.932C>T (p.A282V), in either a homozygous or compound heterozygous state, is associated with this “mild” form of IVA [30]. However, the long-term outcome of screened individuals with different types of IVA still needs to be defined.

## 2. IVA Newborn Screening: Diagnosis, Birth Prevalence and Differential Diagnosis

Introduction of tandem mass spectrometry (MS/MS) for NBS allowed the detection of elevated levels of C5-carnitine in dried blood spots [43]. In urine, the elevation of IVG confirms the metabolic diagnosis of IVA [44]. The first countries that introduced IVA to their NBS programs were Australia, where IVA was included in the New South Wales NBS program in 1998 [45], and Germany, where it was first included in the Bavarian NBS program in 1999 [8]. Since then, it has been implemented in national NBS programs in about 30 countries worldwide (Table 1), and most recently (2015), in England and Wales. In addition, in the absence of government-run screening programs, NBS for IVA is offered on a private basis in some countries, e.g., South Africa and Lebanon.

Using a data set of 1.6 million newborns from Germany, the birth prevalence of IVA was calculated to be 1 in 67,000 [8]. Prevalences from other countries were reported to be lower, such as 1 in 660,000 in Taiwan [46] or 1 in 105,000 in Portugal [47]. In Australia, the prevalence of IVA has been shown to be 1 in 775,600 in the unscreened population and 1 in 230,750 in a screened cohort [48]. Similarly, an analysis of available evidence by Dionisi-Vici et al. showed a more than four times higher incidence of IVA in the screened population as compared to clinical diagnosis [37], suggesting that the phenotypic spectrum of IVA detected by NBS is different and may include individuals that would not have presented clinically.

Because C5-carnitine represents several isomers, such as isovalerylcarnitine, 2-methylbutyrylcarnitine, or pivaloylcarnitine, elevated levels detected in NBS may account for several differential diagnoses of IVA, including short/branched chain specific acyl-CoA dehydrogenase deficiency (SBCADD) (also called 2-methylbutyryl-CoA dehydrogenase deficiency [2-MBCD] or 2-methylbutyrylglycinuria). SBCADD, an autosomal recessive condition caused by an error in the degradation pathway of l-isoleucine [49], is detected by IVA NBS programs because it shows elevated 2-methylbutyryl (C5)-carnitine, which has the same mass to charge on MS/MS as isovalerylcarnitine [49]. The first patient with SBCADD was reported in 1999 [50]. The *ACADSB* gene structure was described in 2000 [51], and several mutations in this gene have been reported [49,52,53]. In individuals with this disease, urine analysis reveals marked elevations of 2-methylbutyrylglycine [51,54]. Symptoms reported in the literature range from developmental delay, seizures, and autism to neonatal crises [49,55], and protein restriction and supplementation with l-carnitine have been suggested for treatment [49,55,56]. However, most patients seem to be asymptomatic despite metabolic abnormalities. While the frequency of SBCADD was found to be higher and its variability greater following the introduction of MS/MS into NBS programs, the last report focusing on this condition was published in 2013 [49]. There is little information on the long-term clinical outcome of individuals with SBCADD, but overall, this condition is assumed to be benign.

With NBS becoming an important part of pediatric preventive strategies worldwide, several diagnostic pitfalls have come to attention. Pivaloylcarnitine, a derivative of antibiotics containing pivalic acid, can be mistaken for isovalerylcarnitine in NBS blood samples, and treatment of mothers with these antibiotics before delivery has been blamed for a number of false positive NBS results [57,58,59,60,61]. Sivelestat, a neutrophil elastase inhibitor used to treat acute respiratory distress syndrome, also contains pivalic acid and can lead to false positive NBS results for IVA [62]. In order to differentiate SBCADD from IVA and to exclude interference from antibiotics, urine acylglycine analysis and/or quantitative organic acid analysis are performed. Furthermore, several strategies for second-tier testing in dried blood spots have been developed, including stable isotope dilution MS/MS analysis to determine isovalerylglycine [63,64] and ultra-performance liquid chromatography (UPLC)-MS/MS analysis of C5-carnitines [59,65].

## 3. Emerging Spectrum of the Disease

### 3.1. Clinical Presentation

Today, an increasing number of patients are diagnosed by NBS using MS/MS before the onset of symptoms. A large study analyzing 1.6 million newborns from Germany found that nearly half of the cases detected by NBS (11/24) were defined as “metabolically mild or intermediate” [8]. It may be assumed that individuals with the mild phenotype identified by NBS may remain asymptomatic throughout their lives [30]. This hypothesis is supported by family studies identifying asymptomatic individuals with biochemical evidence of IVA and genotypes identical to their younger siblings identified by NBS [30] and by the reported increase in the prevalence of organic acidurias in screened cohorts as compared to clinical ascertainment [32,37,48,66,67].

Potentially life-threatening episodes of metabolic acidosis associated with lethargy or impaired consciousness—often but not always following situations of catabolic stress—are common in IVA. The first life-threatening catabolic episode in patients with the acute form usually occurs by the end of the first week of life (“acute neonatal form”) [38,39,66]. Depending on the time of NBS sampling and turnaround time, patients with this form may even present clinically before the NBS result is reported [32]. Early symptoms are nonspecific. Newborns are feeding poorly and present with emesis often associated with dehydration, lethargy, and sometimes seizures [39,66,68]. Metabolic acidosis with an elevated anion gap representing the accumulation of organic acids, secondary hyperammonemia, and hyperglycemia or hypoglycemia are reported as laboratory findings [39,66,67]. An unpleasant smell of “sweaty feet” is characteristic for IVA and can be noticed in acutely sick infants [39], but may not be recognizable in otherwise well patients. If left untreated, the clinical condition can worsen to coma and ultimately death [39]. The incidence of acute life-threatening catabolic episodes is highest in early infancy and decreases with age. Still, patients may become sick later in childhood with intermittent bouts of illness with vomiting and metabolic acidosis [39,66,67], often precipitated by infections or other physiologic stressors [39]. Interestingly, in a study of 21 children with symptomatic IVA, no such event was observed after nine years of age [39], although acute metabolic decompensations have been reported in adult life [69,70].

Apart from life-threatening metabolic crises, unscreened patients may first present later in life with neurological symptoms and cognitive impairment (“chronic intermittent presentation”) [39,66]. Symptoms are often nonspecific and include feeding difficulties, vomiting, and failure to thrive and/or developmental delay and cognitive impairment. Neurological manifestations of IVA relate to EEG abnormalities or seizures and motor dysfunction [71,72]. Similar to findings in other organic acidurias [73], there have been single reports of patients with pancreatitis [39,74,75,76]. Liver fibrosis [28] and, most recently, optic nerve atrophy [71] have also been associated with IVA. Pregnancies of affected women have been reported as uneventful [77,78,79].

### 3.2. Management/Treatment

Long-term treatment strategies aim to: (1) reduce the production of toxic metabolites by the restriction of protein or leucine intake; and to (2) enhance the conjugation of potentially toxic free isovaleric acid to its non-toxic conjugates isovalerylcarnitine and isovalerylglycine, which are excreted by the kidneys via supplementation of l-carnitine and/or l-glycine [44,80,81].

Many patients with a clinically symptomatic type of IVA follow a protein-restricted diet to reduce their intake of leucine and limit the production of toxic metabolites. In order to cover age-appropriate amounts needed for normal growth and to avoid malnutrition, leucine-free amino acid supplements enriched with micronutrients may be needed. However, wide differences in actual dietary practices in IVA have been documented [82]. l-carnitine is usually given at a dosage of 100 mg/kg × day in three doses. Because it remains unknown if subjects with the mild IVA phenotype detected by NBS might experience metabolic crises or long-term neurological manifestations, these individuals may be advised to take l-carnitine, although it is unclear whether it prevents metabolic crises. A low dosage of 30 to 50 mg/kg × day has been proposed for these individuals [44]. l-glycine may be omitted from long-term treatment, especially in individuals with the mild phenotype. If given, the dosage is usually between 100 and 300 mg/kg × day in three doses [56].

During intercurrent illness, the production of isovaleryl-CoA might be increased due to a higher rate of breakdown of the endogenous protein. Therefore, the prevention of catabolic episodes is crucial. Anabolizing measures including oral glucose polymer solutions or high-dose glucose and potentially lipid infusions may be necessary to secure an adequate energy supply [44,83]. A short-term decrease of protein intake should also be part of the acute treatment protocol [44,83,84]. In order to prevent the accumulation of toxic metabolites, increased doses of l-carnitine (up to 400 mg/kg × day) [83,84] and l-glycine (by 50% to 100%) [85] have been recommended. As for other classic organic acidurias, treatment with *N*-carbaglutamate has been suggested for the treatment of acute neonatal hyperammonemia in IVA [86]. As it cannot be entirely excluded that periods of illness might trigger unfavorable effects in patients with the mild type of IVA, individuals with this condition should also be counseled to follow an emergency protocol and to increase the intake of l-carnitine and energy during febrile illnesses.

### 3.3. Outcome

Mortality is highest in patients with an early clinical onset: an analysis of 155 published patients with symptomatic IVA showed a mortality rate of about one-third during the initial metabolic crisis in patients diagnosed within the first five weeks of life, whereas patients diagnosed thereafter had a low mortality rate of only 3% [39]. Patients with an early presentation who died during the initial catabolic episode had a significantly earlier onset of symptoms than patients who survived this initial catabolic episode.

Early initiation of treatment in IVA, i.e., starting therapy during the first weeks of life, was shown to decrease the frequency of severe ketoacidotic crises and was associated with an overall good clinical outcome [85]. Patients diagnosed by NBS often appear asymptomatic [87,88], and early diagnosis of IVA has been reported to correlate with a good neurocognitive outcome: an extensive review of published patients found that in patients diagnosed in the first five weeks of life, 85% had an unremarkable neurocognitive outcome as opposed to only 45% of patients who were diagnosed after the fifth week of life [39]. Similarly, in a South African population, all patients diagnosed within the neonatal period, but only 43% of patients diagnosed thereafter, had a normal neurocognitive outcome [67].

However, not all studies available to date appear to support the relevance of early diagnosis: an analysis of 52 patients from the European Registry and Network for Intoxication Type Metabolic Diseases (E-IMD) showed a statistical trend for normal development in patients diagnosed by NBS as compared with patients who were diagnosed after the onset of symptoms. This trend disappeared after the omission of patients with the “mild” phenotype from the analysis [38]. Cognitive function in a series of 16 Spanish patients was shown to be within the normal range in both patients diagnosed clinically and patients detected by NBS [32]. Overall, neurological sequelae and organ manifestations in IVA have been shown to be less common as compared with other classic organic acidurias [39,71,89].

Whether patients with a biochemically mild type of IVA may develop any clinical symptoms under certain circumstances remains open, since long-term data on the outcome of individuals diagnosed by NBS are still lacking.

## 4. Conclusions

### IVA Newborn Screening—Outlook and Challenges

The possibility of pre-symptomatic diagnosis through NBS and the apparent benefit that has been demonstrated for patients diagnosed and treated early [39] make IVA an ideal candidate for NBS programs. An additional “mild” form of IVA with only slight biochemical abnormalities and a potentially asymptomatic phenotype has been discovered by NBS. Still, there is little information on the long-term outcome of patients with this mild type of the disease, and it is not known whether these patients are actually at risk for severe catabolic episodes. With IVA being included as a target disorder of NBS programs in a growing number of countries worldwide, more of these individuals will be identified. Overall, longitudinal studies of screened individuals with IVA are needed to allow for a better understanding of the long-term outcome and clinical spectrum including the “mild” phenotype and to provide the basis for management recommendations and counseling. Results may also allow considering the adjustment of NBS cut-off levels in order to not detect individuals with benign variants.

## Figures and Tables

**Table 1:** Countries with published experience in NBS for IVA.

Region	Country	Local Specifics	IVA Targeted by NBS Since (as Available)	Reference/Source
Asia Pacific	Australia			[5]
	China	No full population screening		[5]
	India	No full population screening; program not government funded		[5]
	Japan			[5]
	Malaysia	No full population screening		[5]
	New Zealand		2006	[6]
	Philippines	No full population screening		[5]
	Singapore			[5]
	South Korea			[5]
	Thailand	No full population screening		[5]
	Taiwan			[5]
Europe	Austria		2002 ^a^	[5]
	Belgium		2009 (Pilot 2007) ^b^	[5]
	Czech Republic		2010 ^c^	[5]
	Denmark		2012	[5,7]
	Estonia	No full population screening		[5]
	Germany		Bavaria 1999 Nationwide 2005	[8,9]
	Greece			[10]
	Hungary			[5]
	Iceland		2008 ^d^	[5]
	Italy	No full population screening		[5]
	Liechtenstein			[5]
	Macedonia	No full population screening	2013 ^e^	Personal communication ^e^
	Netherlands			[5]
	Norway			[5]
	Poland			[5]
	Portugal			[5]
	Russia			[5]
	San Marino	No full population screening		[5]
	Spain			[5]
	Sweden			[5]
	Switzerland			[5]
	United Kingdom	Not in Scotland and Northern Ireland	2015 (Pilot 2012) ^f^	[5]
North America	United States	IVA included in all states but District of Columbia and Massachusetts		[5]
	Canada	IVA included in all provinces/territories but Newfoundland & Labrador; IVA screened by urine in Quebec		[11]
South America	Argentina	Offered exclusively in the private sector		[5]
	Brazil	Offered exclusively in the private sector		[5]
	Chile	Offered as selective screening		[5]
	Colombia	No full population screening; offered in the private sector		[5]
	Costa Rica			[5]
	Dominican Republic	Offered exclusively in the private sector		[5]
	Mexico	No full population screening		[5]
	Uruguay	No full population screening		[5]
	Venezuela	Offered exclusively in the private sector		[5]
Africa	South Africa	Offered exclusively in the private sector		[12]
Middle East	Kuwait		Pilot 2004–2006	[13] Personal communication ^g^
	Lebanon	Offered exclusively in the private sector	2006	[14,15]
	Saudi Arabia			[15]
	Qatar		2004	[15,16]
	United Arab Emirates		2011	[17]

IVA: isovaleric acidemia; NBS: newborn screening; ^a^ Personal communication by Maximilian Zeyda; ^b^ Personal communication by François Boemer; ^c^ Personal communication by Viktor Kožich; ^d^ Personal communication by Leifur Franzson; ^e^ Personal communication by Violeta Anastasovska; ^f^ Personal communication by Jim Bonham; ^g^ Personal communication by Laila Bastaki.
